# Exploring Redox–Inflammation Interactions: Tanshinones from *Salvia miltiorrhiza* as Potential Modulators of Intestinal Health in Livestock

**DOI:** 10.3390/antiox15101289

**Published:** 2026-10-04

**Authors:** Mahmoud Soliman, Cheng Du, Xinyu Liu, Liangwenyao Zhao, Linli Cheng

**Affiliations:** 1 College of Veterinary Medicine, China Agricultural University, Beijing 100084, China; 2 Department of Animal Production, Faculty of Agriculture, Kafrelsheikh University, Kafr El Sheikh 35111, Egypt

**Keywords:** tanshinones, *Salvia miltiorrhiza*, redox–inflammation crosstalk, intestinal barrier, phytogenic feed additives, livestock

## Abstract

Tanshinones from *Salvia miltiorrhiza* have been studied in biomedical systems, and a growing intestinal literature includes porcine epithelial-cell models and experimental colitis. This integrative narrative review summarizes intestinal findings separately from evidence in non-livestock models and from mechanistic extrapolation. Direct food-animal evidence for purified tanshinones as dietary additives remains limited; studies of whole-plant preparations and sodium tanshinone IIA sulfonate are not equivalent to feed trials of native tanshinones. Reported effects involve redox and inflammatory signaling, epithelial barrier-related measures, and intestinal injury models but do not establish species-specific efficacy, dose response, safety, or performance benefits. Microbiota–metabolite–immune relationships provide a rationale for future testing, not evidence that tanshinones improve livestock microbiota or production. Key translational gaps include dose escalation, pharmacokinetics, feed-processing stability, species-specific toxicology, residues, withdrawal periods, and interactions. The available evidence supports testable research questions rather than a validated feed-additive recommendation.

## 1. Introduction

The progressive global restriction on antibiotic growth promoters has created an urgent need for mechanistically rational and sustainable alternatives that support intestinal health and performance in intensive livestock systems [1,2,3,4]. Simultaneously, the recognition that oxidative stress and chronic low-grade inflammation are central, interconnected drivers of gut barrier dysfunction, dysbiosis, and reduced productivity in pigs, poultry, and ruminants has shifted attention toward compounds capable of modulating the redox–inflammation axis [5,6,7,8,9,10,11].

*Salvia miltiorrhiza* Bunge (Danshen) and its abietane diterpenoids, the tanshinones, have been studied in biomedical pharmacology [12,13,14,15,16,17,18,19]. Tanshinone IIA-related NF-κB and iNOS findings have been reported in experimental systems [20,21,22], but these results should not be read as evidence of livestock intestinal efficacy. Intestinal studies now include porcine epithelial-cell models and experimental colitis, alongside limited piglet evidence for an intravenously administered water-soluble derivative and studies of complex *S. miltiorrhiza* preparations. These evidence categories are distinct. This integrative narrative review summarizes direct intestinal findings separately from non-livestock models and mechanistic extrapolation; it does not claim a formal systematic-review process or a validated feed-additive application.

Literature search and evidence selection. This integrative narrative review was informed by targeted searches of PubMed/PMC records and publisher websites, with citation checking of relevant primary studies and reviews, through 28 September 2026. Queries combined the compound names (“tanshinone IIA,” “cryptotanshinone,” and “*Salvia miltiorrhiza*”) with intestine/gut, cell-model, colitis, livestock, piglet, poultry, redox, inflammation, and barrier-related terms. Primary studies directly examining intestinal cells or in vivo gut models were prioritized; food-animal studies of plant preparations were included as contextual evidence, while nonintestinal biomedical studies were used only for pathway background. Evidence from mixed herbal preparations was kept distinct from evidence on purified tanshinones. Reviews were used for background, not as substitutes for primary evidence. This was a targeted narrative search, not a registered systematic review: databases were not searched exhaustively, and formal screening, risk-of-bias assessment, and meta-analysis were not performed.

## 2. Chemistry and Pharmacology of *Salvia miltiorrhiza* and Tanshinones

### 2.1. Major Bioactive Constituents and Their Multi-Target Nature

**Figure 1:**
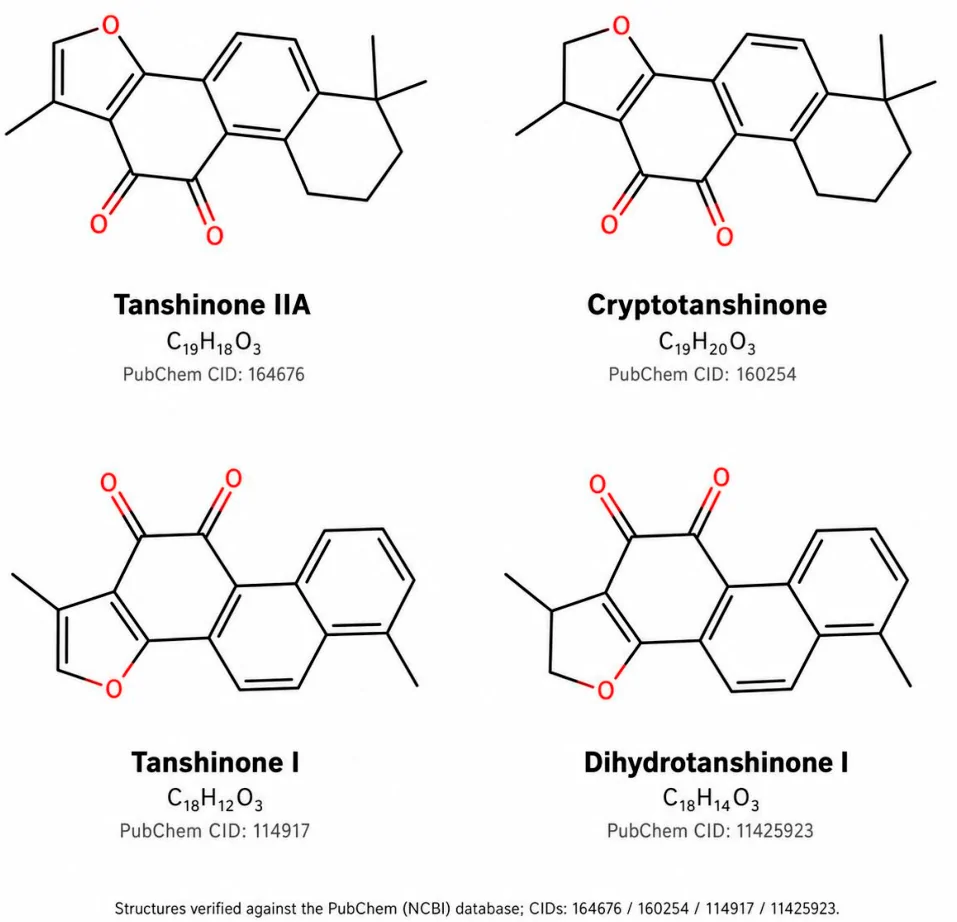
Chemical structures of the principal tanshinones extracted from *Salvia miltiorrhiza*, redrawn from the canonical PubChem (NCBI) deposit. Ring heteroatoms, carbonyl positions, methyl groups, and furan/dihydrofuran rings match the official records. PubChem CIDs: Tanshinone IIA 164676 (C_19_H_18_O_3_); Cryptotanshinone 160254 (C_19_H_20_O_3_); Tanshinone I 114917 (C_18_H_12_O_3_); Dihydrotanshinone I 11425923 (C_18_H_14_O_3_). Structures verified against the PubChem (NCBI) database. The shared abietane scaffold is a structural feature; it does not by itself establish target engagement or biological effect.

The lipophilic fraction of *Salvia miltiorrhiza* contains abietane-type diterpenoids known as tanshinones; redox-related and anti-inflammatory activities have been reported in the pharmacological literature [13,18,23]. Tanshinone IIA, cryptotanshinone, tanshinone I, and dihydrotanshinone I are the most abundant and pharmacologically relevant compounds (Table 1). These compounds are poorly water-soluble; however, varied pharmacological activities have been reported, consistent with multiple cellular targets rather than a single-receptor mechanism [16,17,24]. This breadth could affect multiple cellular processes, but does not establish a beneficial or simultaneous effect on oxidative stress and inflammation; it may also complicate oral exposure and pharmacokinetic optimization for feed delivery. The chemical structures of these principal tanshinones are shown in Figure 1, and their key pharmacological attributes are summarized in Table 1. Intestinal cell and experimental-colitis evidence is summarized in Table 2. 

### 2.2. Pharmacokinetic Considerations for Livestock Application

Native tanshinones present oral-delivery challenges related to limited solubility and reported first-pass metabolism [18,33]. Sodium tanshinone IIA sulfonate is a water-soluble derivative with clinical use in humans [31], but this does not establish suitability for feed. Candidate delivery strategies, including encapsulation or protected formulations, require evaluation for species-specific exposure, processing stability, efficacy, safety, and cost before livestock use can be considered.

## 3. The Redox–Inflammation Crosstalk: A Central Hub for Gut Health

Oxidative stress and inflammation are coupled through bidirectional molecular crosstalk [34,35,36,37,38,39,40,41]. Reactive oxygen species (ROS) can activate NF-κB, and inflammatory mediators can amplify ROS production; Nrf2-associated responses can also be induced by oxidative signals. Under production stressors, disruption of these processes may contribute to inflammation and barrier dysfunction. In biomedical and cell systems, tanshinones have been associated with altered redox measures, inhibition of kinases linked to NF-κB, and Nrf2-related responses [16,17,20,21,42]. Direct ROS scavenging by tanshinones should not be assumed: the reviewed evidence does not establish that direct radical scavenging explains the reported protective effects. Quinone redox activity may yield context- and concentration-dependent responses, including pro-oxidant effects; ROS generation has been reported in anticancer models [43], but those findings do not establish the intestinal response. Redox direction, concentration dependence, possible hormetic responses, and safety in the rapidly renewing intestinal epithelium therefore require direct testing. These findings provide a rationale for study, not evidence of efficacy in livestock.

### Evolutionary and Functional Conservation of Redox–Inflammatory Nodes Across Biomedical and Livestock Species

A central question in translating tanshinone pharmacology from the biomedical literature to livestock gut health is whether relevant molecular nodes are conserved across species. Core domains and pathway components are conserved across vertebrates, but conservation of protein architecture does not establish conserved pharmacological responses. Pharmacokinetics, feed-matrix effects, microbial metabolism, tissue distribution, and responses to tanshinones must be tested separately in swine, poultry, and ruminants. The Nrf2–Keap1–ARE system is a conserved cytoprotective pathway [44,45,46], and mammalian Keap1 cysteine residues have established roles in general Nrf2 regulation [47,48,49].

Keap1 cysteine sensing is established in general Nrf2 biology [47,48,49], but the cited evidence does not establish direct tanshinone adduction or oxidation of Keap1 cysteines. A Keap1-cysteine mechanism for tanshinone-associated Nrf2 responses therefore remains unproven and should not be presented as the operative route. Conservation of the Nrf2–Keap1–ARE module supports a testable question; it does not demonstrate equivalent compound response in livestock.

The conservation of signaling components provides a basis for hypothesis-driven work, but does not demonstrate conserved tanshinone pharmacology. Which molecular targets are engaged in livestock tissues, at what exposures, and with what outcomes remains to be established. Biomedical evidence can inform candidate endpoints only when its source and limitations are made explicit.

**Figure 2:**
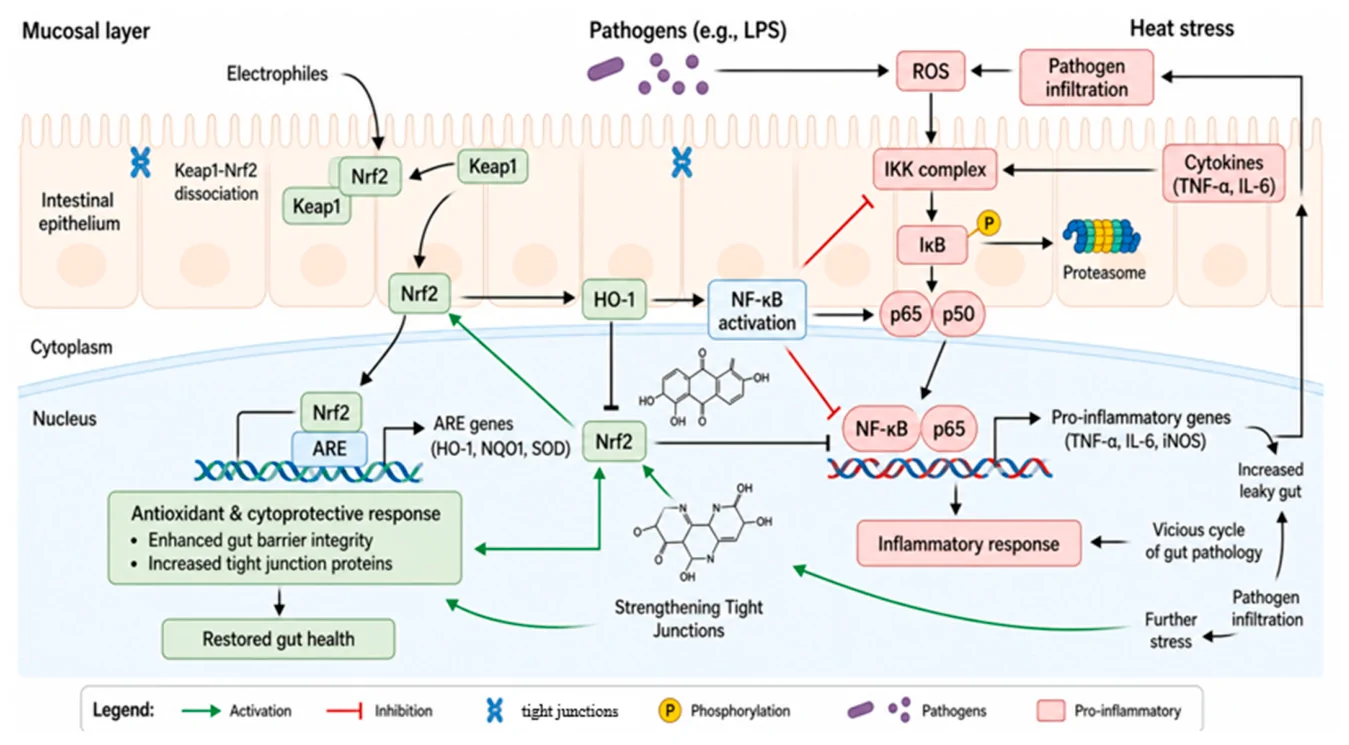
Bidirectional crosstalk between Nrf2-mediated antioxidant defense and NF-κB-driven inflammation. The arrows summarize proposed pathway relationships; they do not establish tanshinone-specific target engagement or efficacy in livestock intestinal tissue. In the legend, green arrows indicate activation, red T-bars indicate inhibition, blue symbols mark tight junctions, yellow circles indicate phosphorylation, purple shapes/dots represent pathogens, and pink boxes indicate pro-inflammatory elements.

The proposed bidirectional crosstalk between Nrf2-mediated antioxidant defense and NF-κB-driven inflammation is summarized in Figure 2.

**Table 2:** Structured summary of intestinal evidence relevant to tanshinones, separated by compound/preparation and evidence source.

Compound/Preparation and Study	Model/Species	Reported Finding/Outcome	Evidence Level and Limitation	Ref.
Tanshinone IIA + DON	IPEC-J2 porcine jejunal epithelial cells.	DON-associated oxidative injury, barrier disruption, mitochondrial dysfunction and cell injury were attenuated in vitro.	Direct porcine intestinal-cell evidence; not an in vivo feed trial.	[25]
	IPEC-J2 porcine jejunal epithelial cells.	DON-associated oxidative stress, inflammatory signaling and NLRP3-related pyroptosis were attenuated in vitro.	Direct porcine intestinal-cell evidence; not a production-animal efficacy trial.	[26]
Tanshinone IIA	DSS colitis, mice.	Colitis outcomes and inflammatory signaling were studied in relation to PXR.	Direct intestinal in vivo evidence in a non-livestock model.	[27]
	DSS colitis, mice and isolated neutrophils.	Neutrophil infiltration/activation and inflammatory outcomes were examined.	Direct intestinal in vivo evidence in a non-livestock model plus in vitro cell evidence.	[28]
	TNBS chronic colitis, mice; primary intestinal cells.	Nrf2-dependent changes in ferroptosis/oxidative injury were examined.	Direct intestinal in vivo evidence in a non-livestock model.	[29]
	AOM/DSS colitis-associated colorectal tumorigenesis, mice.	Intestinal permeability, inflammatory outcomes, and tumorigenesis were assessed after intraperitoneal tanshinone IIA.	Direct intestinal in vivo evidence in a non-livestock model; route and disease model do not establish dietary livestock efficacy.	[30]
	IEC-6 intestinal epithelial cells; RSL3 model.	Cellular ferroptosis-related responses were studied.	In vitro intestinal-cell evidence; not livestock.	[50]
Sodium tanshinone IIA sulfonate	Healthy newborn piglets; intravenous administration.	Mesenteric perfusion/hemodynamics were measured.	Direct piglet evidence for an intravenous derivative, not feed-additive efficacy or native tanshinone dosing.	[32]
*S. miltiorrhiza* ultrafine powder	Aged laying hens; dietary preparation.	Laying performance, follicular development, and egg quality were studied; tanshinone IIA was nominated through network/docking analyses.	Direct livestock evidence for a complex plant powder; in silico nomination does not establish tanshinone-specific effects.	[51]
*S. miltiorrhiza* preparation	Laying hens; dietary botanical study.	Laying performance, egg quality, and antioxidant status were evaluated.	Whole-plant preparation, not purified tanshinone; no intestinal efficacy claim is inferred.	[52]
*S. miltiorrhiza* + Isatidis Radix herbal residues	Late-phase laying hens; dietary residue mixture.	Production performance and cecal microbiota outcomes were reported.	Direct livestock evidence for a multi-herb residue mixture; effects cannot be attributed to tanshinones or *S. miltiorrhiza* alone.	[53]
*S. miltiorrhiza* ultrafine powder	Late-laying hens; dietary preparation.	Ileal morphology/barrier and inflammatory measures were reported; tanshinone IIA was nominated through network/docking analyses.	Direct livestock evidence for a complex plant preparation; not proof of tanshinone-specific effects.	[54]
Tanshinone IIA-loaded nanoparticles + neural stem cells	Pig ischemic-stroke model.	Gut homeostasis was assessed under a combined intervention.	Pig model, but combined therapeutic intervention and non-nutritional context preclude attribution to a feed additive or tanshinone alone.	[55]

## 4. Intestinal Barrier Function and the Redox–Inflammation Axis in Livestock

The intestinal mucosal barrier is a dynamic, multilayered structure important for nutrient absorption, immune homeostasis, and exclusion of luminal pathogens and toxins [56,57,58]. In farm animals, it is challenged by weaning, stocking density, heat stress, and feed-borne contaminants [9,10,59,60,61,62,63]. These stressors can affect redox signaling, inflammatory activation, tight-junction proteins, mucus, and microbial communities. Host–microbiota–immune relationships are bidirectional: microbial composition and metabolites, including SCFAs, can influence epithelial and mucosal immune functions, while inflammation and barrier disruption can alter the luminal environment [64,65,66]. A broader review discusses microbiota–immune modulation in disease contexts [67], but does not establish tanshinone effects or livestock intestinal efficacy. This general biology supports measuring microbial, metabolite, immune, and barrier endpoints together; it is not evidence that tanshinones cause microbiota-mediated recovery.

**Figure 3:**
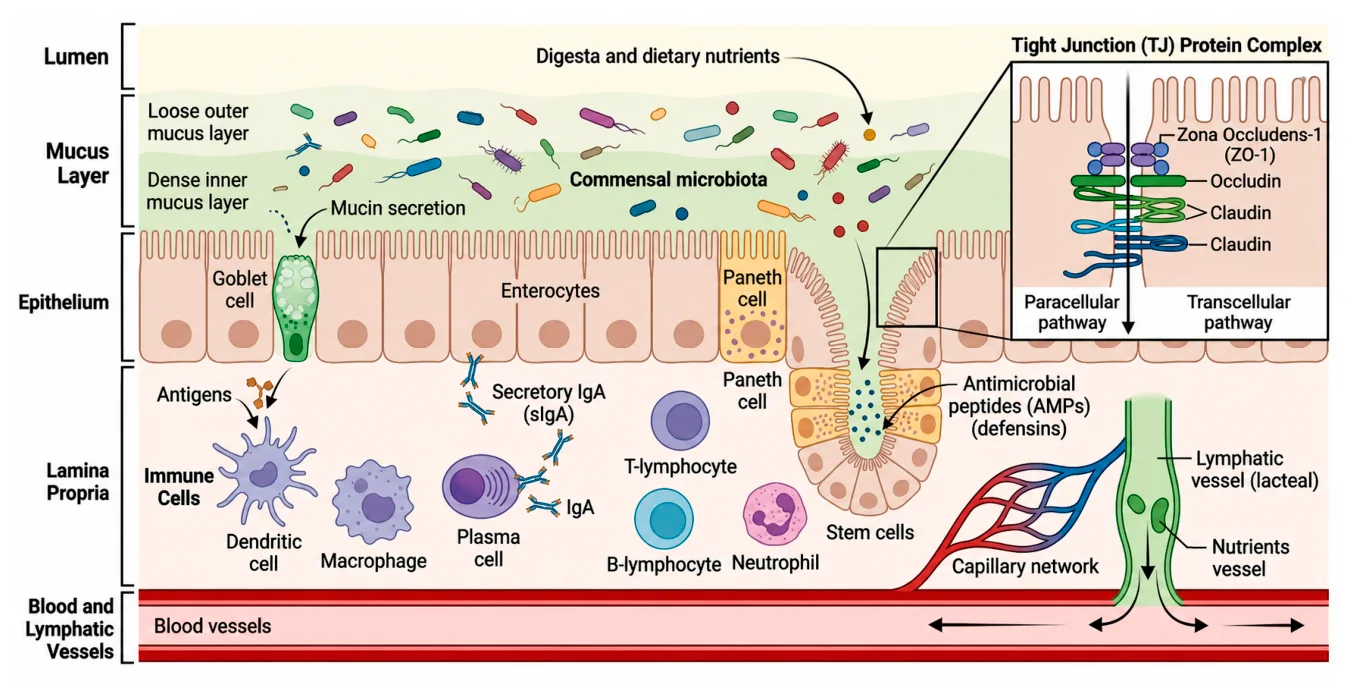
Multi-layered architecture of the intestinal mucosal barrier in livestock and the principal redox–inflammation insults that compromise it under commercial production conditions. Created with BioRender.com. Arrows show the directions of the illustrated transport or signaling processes; colors and icons distinguish anatomical layers, cell types, microbes, and vascular structures. Table 3 summarizes major livestock barrier vulnerabilities, associated production stressors, and redox–inflammation consequences.

The multilayered intestinal barrier and the principal production-related redox–inflammation insults are illustrated in Figure 3.

**Table 3:** Intestinal barrier vulnerabilities, common production stressors, and associated redox–inflammation consequences in livestock.

Vulnerability Point	Stressor in Livestock	Redox–Inflammation Consequence	References
Tight junctions	Weaning, heat, density.	ROS up → NF-κB up → ZO-1/occludin/claudin down → permeability up.	[56,57,68]
Mucus layer and goblet cells	Oxidative damage, inflammation.	Mucin depletion reduces protection.	[9,60]
Microbiota–SCFA axis	Dysbiosis post-weaning or heat stress.	Downregulated butyrate producers → weakened barrier and anti-inflammatory tone.	[10,64]
Enterocyte redox balance	High metabolic demand plus external stressors.	Mitochondrial ROS, lipid peroxidation, and apoptosis.	[7,8]

Note: Arrows (→) indicate downstream biological consequences and pathway progression.

## 5. Phytogenic Additives and Oxidative Stress in Production Systems: The Current Landscape

### 5.1. Phytogenics as Antibiotic Alternatives: Strengths and Limitations

A wide range of plant-derived compounds, including essential oils, polyphenols, flavonoids, and saponins, have been evaluated as alternatives to antibiotics [1,69,70,71,72,73,74,75,76,77]. Their reported effects include antimicrobial, antioxidant, anti-inflammatory, and microbiota-related outcomes [2,3,78]. Because many products are mixtures, constituent-specific contributions and dose–response relationships can be difficult to assign; this does not establish that purified tanshinones are more efficacious or predictable. Human-focused reviews describe microbiota-dependent polyphenol biotransformation and proposed links between polyphenols, gut microbes, and inflammatory signaling [79,80]. These data provide dietary context only, not direct evidence for tanshinones or livestock feed use.

**Figure 4:**
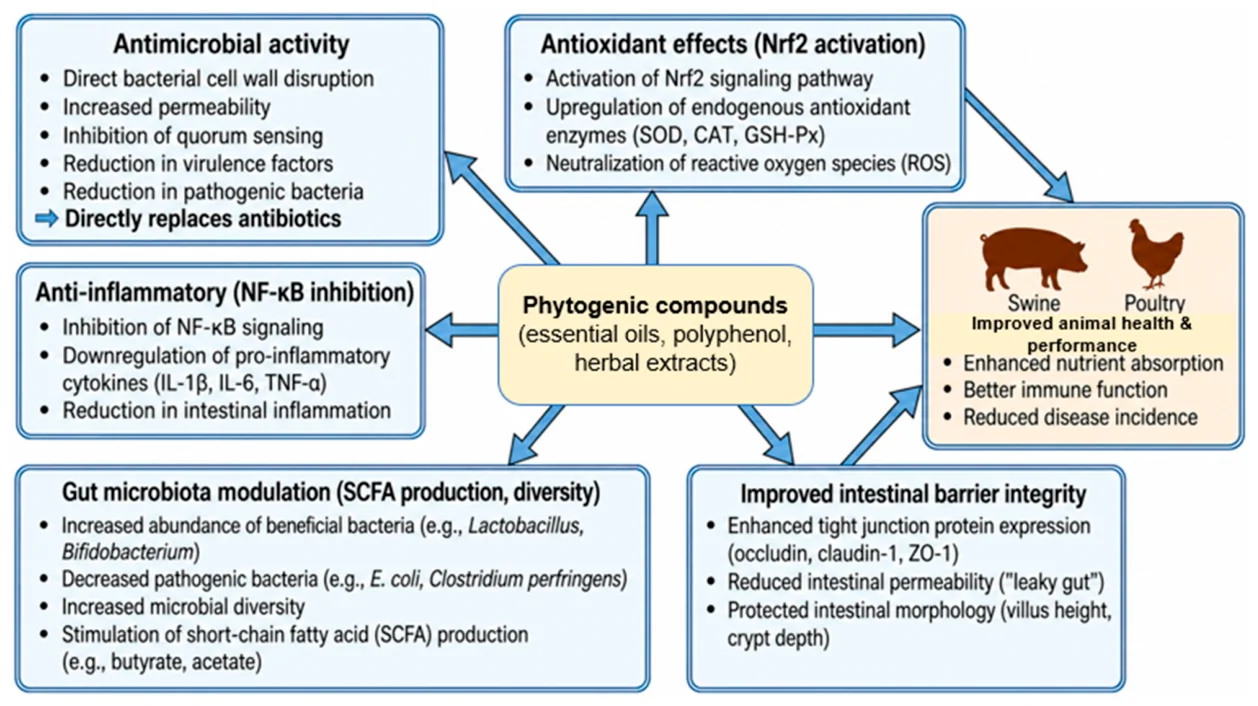
Various mechanisms through which current phytogenic feed additives may operate. The figure does not imply that tanshinones have greater target specificity or efficacy than other phytogenics.

Examples of proposed mechanisms through which current phytogenic feed additives may operate are summarized in Figure 4.

### 5.2. Oxidative Stress as a Pervasive Production Constraint

**Figure 5:**
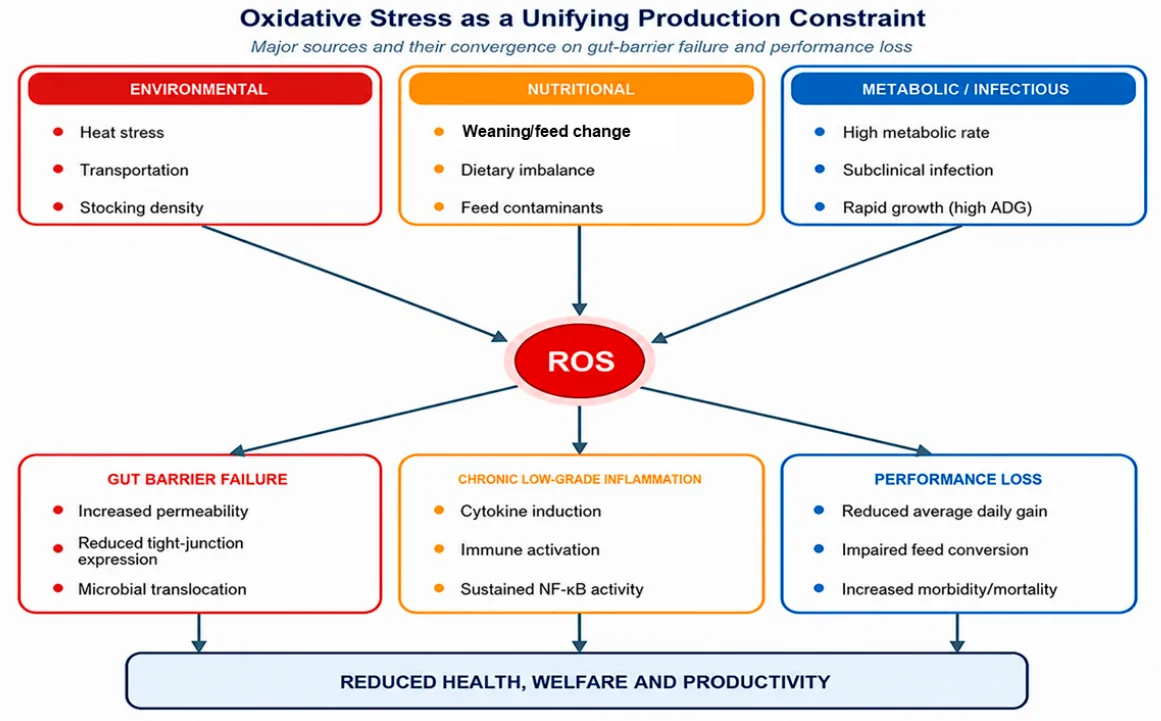
Major sources of oxidative stress in intensive livestock production and their convergence on gut-barrier failure and performance loss. Environmental, nutritional, and metabolic/infectious stressors converge on a shared ROS-production hub that drives gut-barrier dysfunction, chronic low-grade inflammation, and performance loss, ultimately reducing health, welfare, and productivity.

Oxidative stress is recognized as a unifying mechanism underlying many of the performance and health problems encountered in modern livestock systems [5,6,81,82,83]. Sources include high metabolic rates, environmental heat, weaning, transportation, and subclinical infections. The consequences of these changes are impaired barrier function, chronic inflammation, reduced feed efficiency, and compromised immunity, which are among the outcomes that phytogenic interventions have been investigated to mitigate [7,8]. The convergence of major production stressors on gut-barrier failure and performance loss is summarized in Figure 5.

## 6. Tanshinones and Intestinal Health in Livestock: Evidence and Research Needs

Direct intestinal literature includes tanshinone IIA studies in porcine IPEC-J2 cells exposed to DON, addressing mitochondrial quality control, barrier-related measures, oxidative injury, inflammatory signaling, and pyroptosis [25,26]. Tanshinone IIA has also been examined in mouse intestinal models involving PXR-related DSS colitis, neutrophil responses, chronic colitis redox/ferroptosis, and AOM/DSS-associated tumorigenesis [27,28,29,30], as well as in an IEC-6 cell ferroptosis model [50]. In newborn piglets, an intravenous study of sodium tanshinone IIA sulfonate measured intestinal hemodynamics [32]. This derivative study is not a dietary trial. A 2026 laying-hen study of *S. miltiorrhiza* ultrafine powder reported ileal outcomes, while tanshinone IIA was nominated by network and docking analyses; the feed preparation was not purified tanshinone [54]. Other laying-hen studies examined dietary *S. miltiorrhiza* preparations and a mixture of *S. miltiorrhiza* and Isatidis Radix residues, reporting production, antioxidant, egg-quality, or cecal-microbiota outcomes [51,52,53]. These are whole-plant or mixed-residue interventions and do not establish tanshinone-specific effects. A pig ischemic-stroke study used tanshinone-loaded nanoparticles together with neural stem cells, so its gut outcomes cannot be attributed to a tanshinone feed additive alone [55]. Collectively, these studies expand the intestinal evidence base but do not establish dietary efficacy, dose response, or safety of purified tanshinones in production animals. The focus on tanshinones here is analytical, not a claim that they are preferable to salvianolic acids or standardized whole-plant preparations; a purified constituent can support attribution in future experiments, but no head-to-head livestock evidence establishes a preferred candidate.

### 6.1. Evidence Context and Comparators

The redox–inflammation–barrier framework is also used to study other phytogenic and microbiota-directed strategies. No comparative potency, efficacy, EC50/IC50, in vivo effect-size, dose-response, or cost data establish tanshinones as superior, more target-specific, effective at lower inclusion rates, or more predictable than other additives. Human-focused reviews describe variation in polyphenol biotransformation by gut microbial metabotypes and discuss microbiota–polyphenol–inflammation relationships [79,80]; these are context only, not evidence of tanshinone efficacy in livestock. A human clinical-trial meta-analysis of probiotics cannot validate livestock use or predict tanshinone combinations [84]. Any microbiota-directed strategy should be evaluated in a strain-, host-, dose-, and study-design-specific manner; combinations require their own testing.

### 6.2. The Proposed Mechanistic Model for Livestock

**Figure 6:**
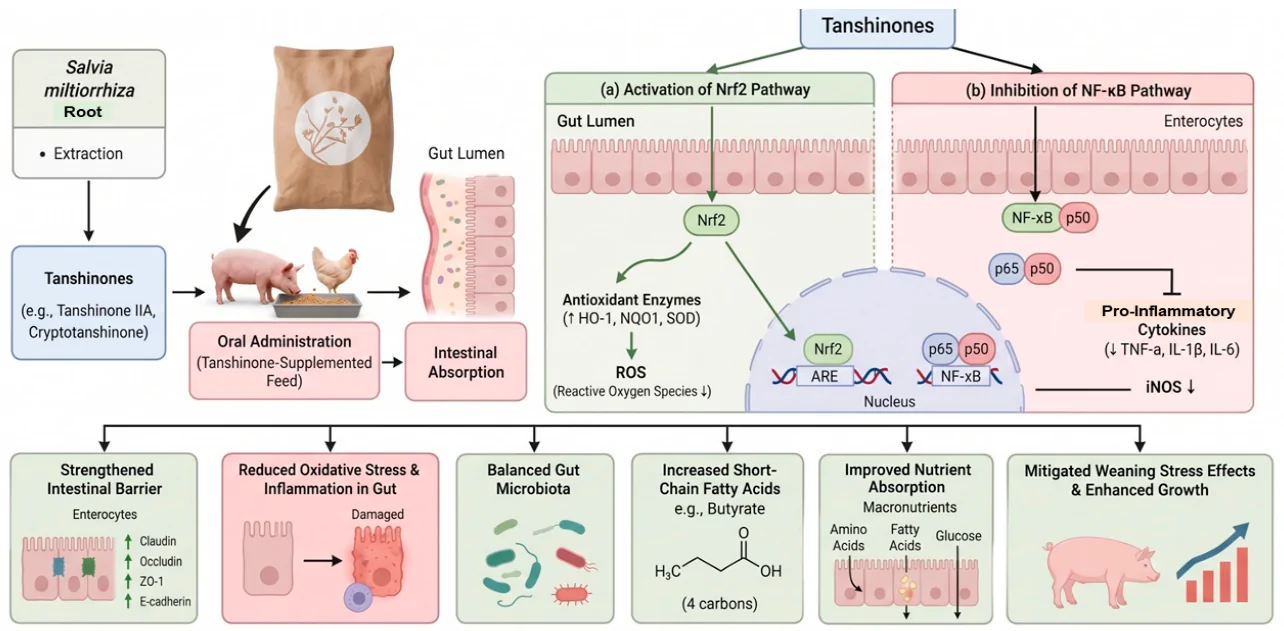
Proposed mechanism of tanshinones from *Salvia miltiorrhiza* in redox–inflammation crosstalk and intestinal health. All downstream effects shown, including microbiota, nutrient absorption, and performance outcomes, are hypotheses requiring direct testing; they are not established effects of tanshinone feed additives in livestock. Arrows and inhibitory bars indicate proposed pathway relationships; the green and red panels distinguish the Nrf2-associated and NF-κB-associated pathways, respectively.

The model in Figure 6 is a hypothesis-generating framework. Candidate endpoints include Nrf2-associated responses, NF-κB/inflammatory measures, epithelial barrier integrity, enterocyte injury, microbiota composition/function, and SCFAs. Evidence comes from distinct models: porcine intestinal-cell studies provide direct in vitro evidence under DON challenge; colitis studies provide intestinal evidence in non-livestock models; and livestock studies of *S. miltiorrhiza* preparations do not isolate tanshinones. Improved resilience, feed efficiency, mortality, or product quality has not been demonstrated for dietary purified tanshinones in food-producing animals. Future studies should use species-specific dose escalation and prespecified endpoints, including barrier function, histology, inflammatory and redox measures, microbiota and SCFAs, and relevant production outcomes. The available literature does not support specifying expected effect sizes or feed doses in advance.

Hypotheses, evidence sources, and candidate endpoints are summarized in Table 4. To make future tests falsifiable, each study should prespecify a primary endpoint, relevant comparator, challenge context, sampling times, and analysis plan, and report treatment effects with uncertainty. Dose levels should be selected through species-specific, safety-led escalation; the current evidence does not justify numerical feed doses or expected effect sizes.

## 7. Translational Considerations: Biomarkers, Species Differences, and Formulation Challenges

**Figure 7:**
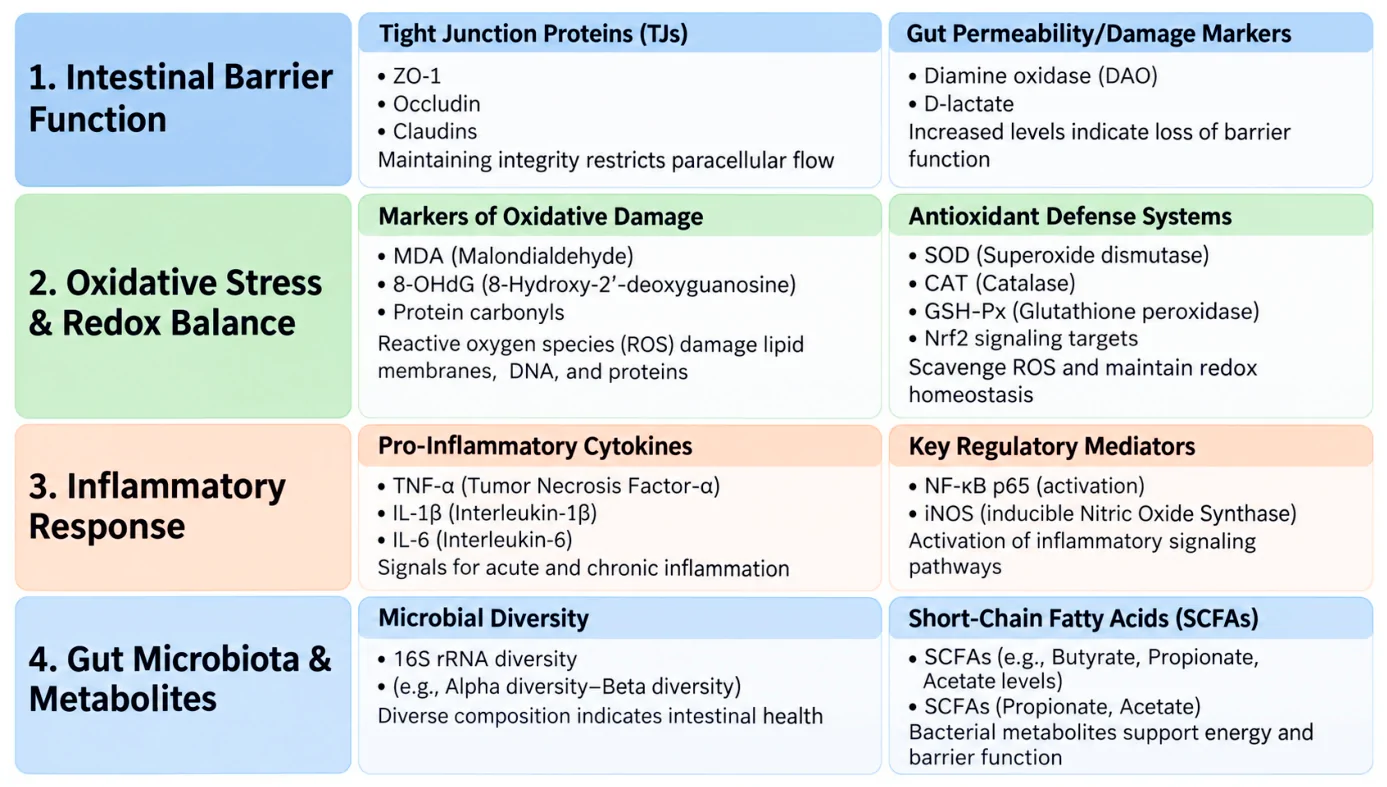
Candidate markers for future assessment of oxidative balance, inflammation, and gastrointestinal outcomes in livestock; validation for tanshinone efficacy is still required.

Future livestock studies should assess efficacy with prespecified, species-appropriate measures of redox status, inflammation, barrier function, and microbiota, alongside exposure and safety. Biomarkers used in pigs and poultry provide candidate measures [7,8,85,86], but their validation for tanshinone treatment and their relation to performance outcomes remain to be established. Candidate markers for assessing oxidative balance, inflammation, and gastrointestinal outcomes are summarized in Figure 7.

### 7.1. Species-Specific Considerations

Pigs and poultry differ in gut anatomy, transit time, and microbiota composition, while ruminants have a pre-gastric fermentation compartment. These differences motivate species-specific testing but do not establish the most responsive species or application point. Weaning stress in piglets and heat/density stress in broilers are candidate contexts for study [10,60,61].

### 7.2. Critical Challenges That Must Be Addressed

Translation requires evidence not currently established for purified tanshinones in food-producing animals. Priorities include species-specific dose escalation and pharmacokinetics; feed-processing and pelleting stability; acute and chronic toxicity; tissue-residue depletion and acceptable residue limits where applicable; evidence-based withdrawal periods; and interaction testing with other feed additives and veterinary therapeutics. Efficacy should be assessed in relevant production contexts, and human pharmaceutical experience should not be treated as a substitute for food-animal data or applicable regulatory review. Costs, sourcing, and standardization also require direct evaluation; no comparative cost advantage is established.

**Figure 8:**
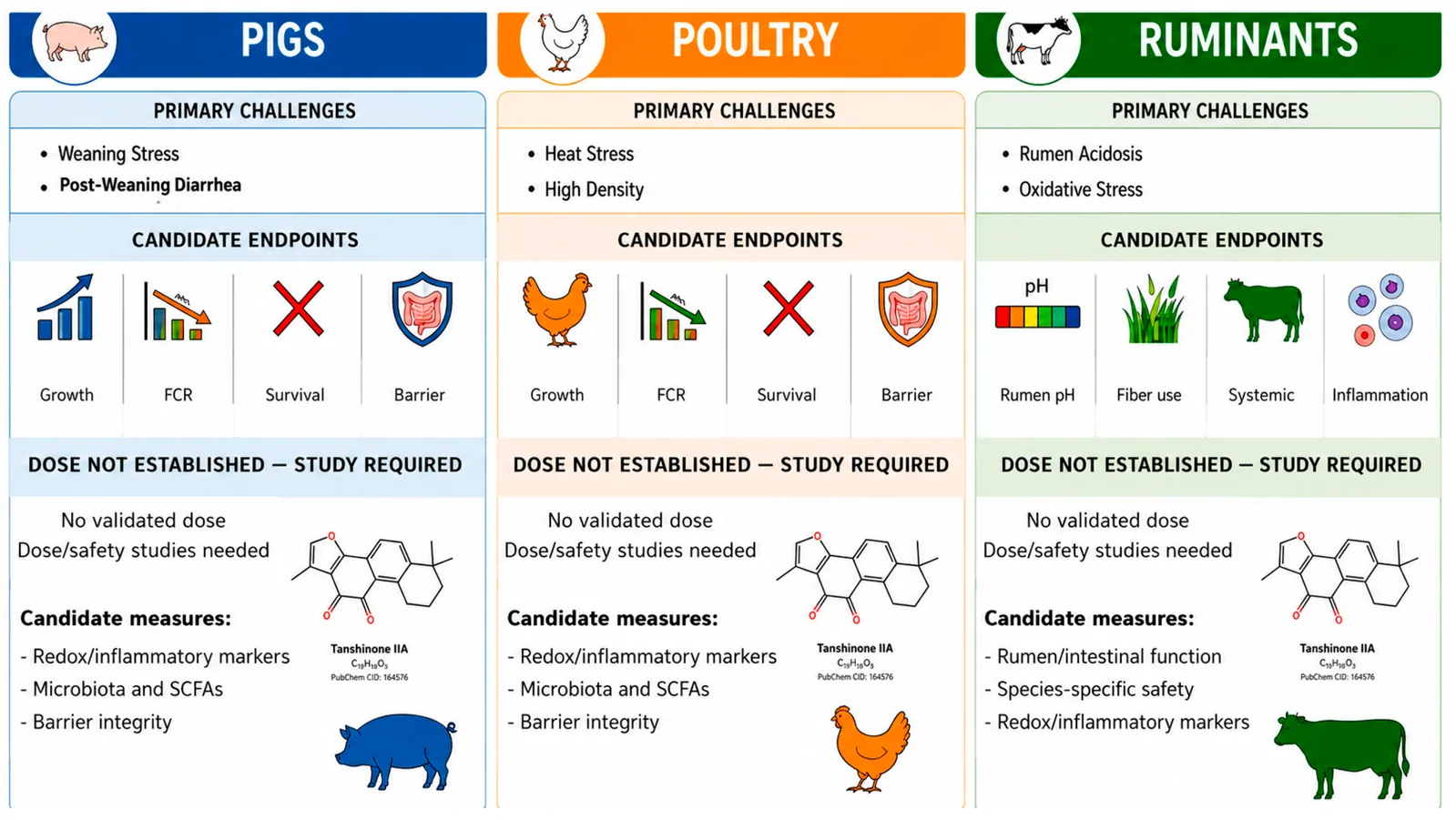
Species-specific considerations and candidate endpoints for future tanshinone studies in livestock. Numerical inclusion levels have been removed because no validated food-animal dose ranges are available. The illustrated endpoints are hypotheses, not demonstrated benefits; dose escalation, efficacy, safety, residues, and withdrawal periods require study. Blue, orange, and green headings identify the pig, poultry, and ruminant panels, respectively; the icons illustrate the listed challenge contexts and candidate endpoints.

Feed-processing stability has not been established for tanshinones. Recovery before and after representative processing conditions should be measured with validated analytical methods; an acceptance criterion should be based on stability, efficacy, and manufacturing data rather than specified in advance. Encapsulation and protected delivery are candidate formulation approaches, but their performance with tanshinones requires testing. Evidence for rumen-bypass delivery of other feed actives [87,88,89] cannot be assumed to apply to tanshinones; rumen stability and post-ruminal release require direct evaluation before ruminant applications are proposed. Species-specific considerations and candidate endpoints for future tanshinone studies are summarized in Figure 8.

**Figure 9:**
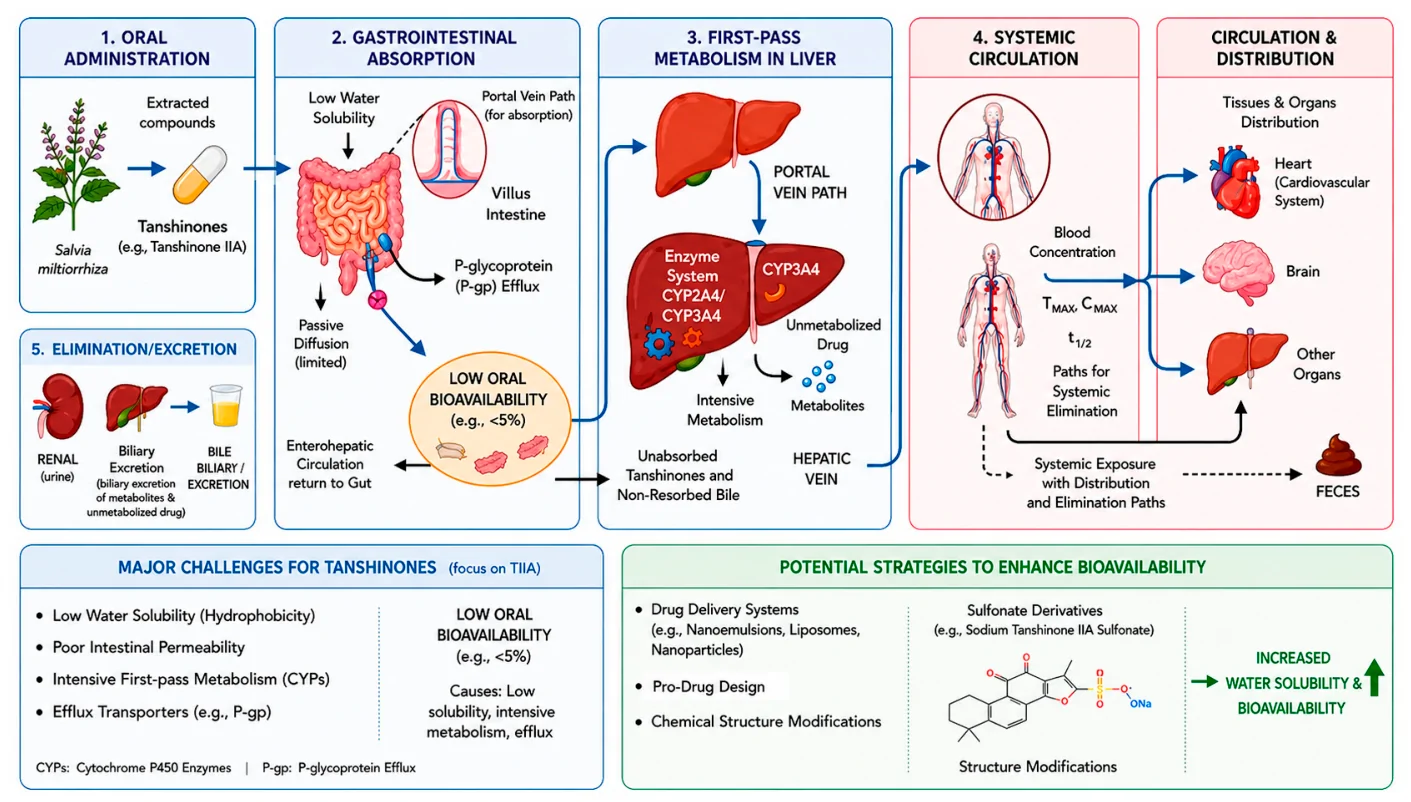
Pharmacokinetic considerations for oral delivery in livestock. Limited solubility and reported first-pass metabolism complicate oral delivery in the biomedical literature [18]; the schematic is not a species-specific livestock pharmacokinetic profile. Formulation approaches, including sulfonated derivatives and encapsulation, are candidates for evaluation [15,31]; their suitability, stability, exposure, and release must be tested in each target species, for which tanshinone pharmacokinetic data remain unestablished. The numbered panels 1–5 depict the sequence from oral administration through absorption, first-pass metabolism, systemic circulation/distribution, and elimination/excretion; arrows indicate the pathways shown.

The pharmacokinetic and oral-delivery considerations requiring evaluation are illustrated in Figure 9.

## 8. Conclusions and Research Priorities

**Figure 10:**
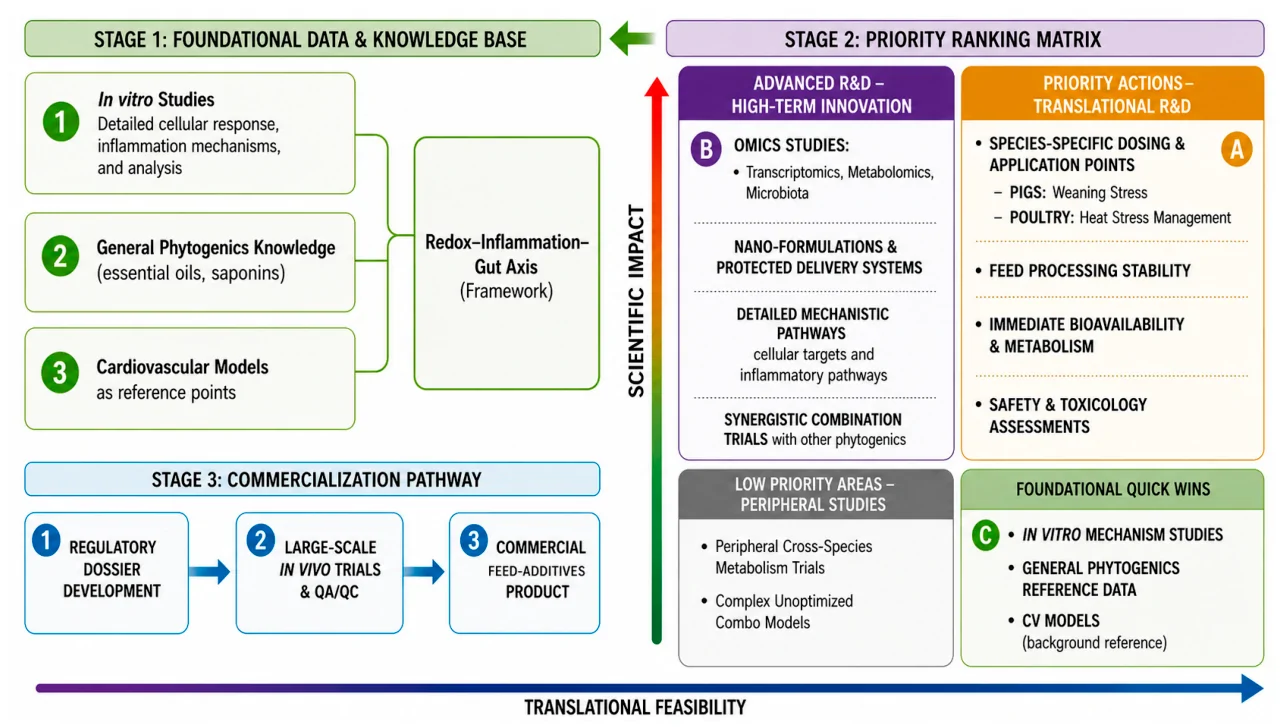
Research roadmap for evaluating tanshinones as potential feed additives. Priorities include foundational species-specific data, safety, bioavailability, and feed-processing stability; commercial translation remains contingent on evidence from adequately designed studies. Numbers identify the listed steps within the roadmap stages, while letters A–C identify the Stage 2 priority categories; the colored panels distinguish the labeled workstreams, and arrows indicate the depicted progression and feasibility/impact axes.

The available evidence includes tanshinone IIA findings in porcine intestinal epithelial-cell models and experimental colitis, a piglet study of intravenous sodium tanshinone IIA sulfonate, and livestock studies of complex *S. miltiorrhiza* preparations. These findings are not interchangeable and do not establish dietary efficacy of purified tanshinones in food-producing animals. Redox–inflammation and barrier pathways offer candidate mechanisms and endpoints, not proof that effects observed in biomedical models will occur in livestock. Future work should establish species-specific exposure, dose response, safety, residues, processing stability, and production outcomes before any feed-use recommendation is considered. The staged research roadmap for evaluating tanshinones as potential feed additives is summarized in Figure 10.

## Figures and Tables

**Table 1:** Major tanshinones and their reported pharmacological context, with evidence source and limits.

Compound	Key Pharmacological Attributes Relevant to Redox–Inflammation	References
Tanshinone IIA	NF-κB-related effects and Nrf2-associated responses have been reported in biomedical models; direct radical-scavenging superiority is not established. Intestinal cell and experimental-colitis evidence is summarized in Table 2.	[16,17,20,21,25,26,27,28,29,30]
Cryptotanshinone	Anti-inflammatory/redox-modulating activities are described in the cited pharmacological literature; livestock intestinal efficacy is not established here.	[13,18]
Tanshinone I and dihydrotanshinone I	The cited sources describe pharmacological activity; they do not establish a metabolic-regulator role or livestock gut efficacy.	[18,23]
Sodium tanshinone IIA sulfonate	Water-soluble synthetic derivative with reported clinical use for pulmonary and cardiovascular indications; a newborn-piglet intestinal hemodynamics study is distinct from dietary efficacy of native tanshinones.	[15,31,32]

**Table 4:** Hypotheses and candidate endpoints for future tanshinone studies; these outcomes are not established livestock effects.

Parameter/Hypothesis	Mechanistic Rationale (Proposed)	Evidence Source/Level Currently Available	Endpoints for Future Testing
Tight-junction integrity may be preserved under defined challenge.	Redox/inflammatory pathways may influence junctional organization.	Direct in vitro IPEC-J2 evidence under DON; animal evidence for a complex *S. miltiorrhiza* preparation; no purified tanshinone feed efficacy trial [25,54].	ZO-1, occludin, claudins; permeability; histology, with species-appropriate validated assays.
Oxidative injury may be modulated.	Nrf2-associated responses and mitochondrial/redox effects are candidates; direct ROS scavenging and Keap1 modification are not established as the operative mechanism.	In vitro porcine cells and non-livestock intestinal models; biomedical evidence [25,26,29,50].	ROS/oxidative-damage and antioxidant-response measures, prespecified by the model.
Inflammatory signaling may change.	NF-κB/inflammasome/neutrophil signaling has been examined in specific models.	Porcine cell studies and rodent colitis models; no demonstration of feed efficacy in livestock [26,27,28,29].	Cytokines, NF-κB/inflammasome markers, and relevant immune-cell measures.
Microbiota–SCFA–immune–barrier links may be tested.	Microbial metabolites can participate in bidirectional host–microbiota–immune relationships; tanshinone-mediated changes remain unproven in livestock.	General gut microbiota evidence plus whole-preparation livestock studies, not tanshinone-specific microbiota evidence [54,65,66,79,80].	Microbiota composition/function, SCFAs, mucosal immune markers, and barrier endpoints; test temporal/functional links.
Growth/performance outcomes are hypotheses, not established tanshinone effects.	Improved gut-health measures could be assessed against performance outcomes, but this relationship has not been established for dietary purified tanshinones.	No validated food-animal tanshinone dose–response efficacy evidence identified.	Feed intake, growth/production, feed conversion and health endpoints in adequately powered, species-specific trials.

## Data Availability

Data sharing is not applicable to this article, as no new data were created or analyzed in this review.
